# Depression, Anxiety and Stress among First-year Medical Students in a Tertiary Care Hospital: A Descriptive Cross-sectional Study

**DOI:** 10.31729/jnma.6420

**Published:** 2021-04-30

**Authors:** Priyanka Shah, Alisha Sapkota, Anjeel Chhetri

**Affiliations:** 1 Nepal Medical College and Teaching Hospital, Kathmandu, Nepal; 2 Department of Psychiatry, Devdaha Medical College, Devdaha, Nepal

**Keywords:** *anxiety*, *depression*, *medical students*

## Abstract

**Introduction:**

Medical students are prone to develop stress, anxiety and depression owing to vastness of curriculum, hectic lifestyle, economic burden, and competitiveness of medical field. The study aims to find out the prevalence of depression, anxiety, and stress among first-year medical students.

**Methods:**

A descriptive cross-sectional study was conducted among 91 first-year students of Bachelor of Medicine and Bachelor of Surgery enrolled in a tertiary care hospital using depression, anxiety, and stress-42 scale along with a questionnaire regarding sociodemographic and stressors for their problems. Whole sampling was done and the study was conducted between June and July 2018 after taking ethical approval from the Research and Institutional Review Committee (Reference Number: 57-074/075).

**Results:**

The highest prevalence among undergraduate medical students was found to be anxiety 54 (59.3%), followed by stress 41 (45.1%) and depression 40 (44%).

**Conclusions:**

Almost half of the first-year medical students reported some level of depression, anxiety, or stress. It is important to implement programs in the early years of the medical school from the administrative level to help and identify students suffering from depression, anxiety, and stress.

## INTRODUCTION

Depression is a mental disorder characterized by depressed mood, loss of pleasure, reduced energy and activity, decreased self-esteem, decreased attention with changes in appetite, and sleep disturbances and can even lead to ideas and acts of self-harm and suicide.^1^ Anxiety is characterized by the feeling of tension, nervousness, worried thoughts, and physical changes such as sweating, trembling, and increase blood pressure.^2^ Stress is a physiological and psychological stimulus that causes bodily or mental tension.^3^

Medical education is one of the most competitive fields that create intense stress among students, which harms the learning process, mental and physical health of students. There has been an increase in the number of suicides and dropouts among medical students.^4,5^ There are fewer studies that assess the burden of depression, anxiety, and stress specifically targeting first-year medical students in Nepal. Therefore it is important to have more studies concerning the mental health of medical students in their early years of medical school.

The study aims to find out the prevalence of depression, anxiety, and stress among first-year medical students.

## METHODS

This study is a descriptive cross-sectional study conducted among first-year students of Bachelor of Medicine and Bachelor of Surgery (MBBS) of Nepal Medical College and Teaching Hospital (NMCTH), Kathmandu, Nepal. First-year students were chosen specifically because there are lesser studies in Nepal which target this group. The study was commenced after ethical approval from the Research and Institutional Review Committee (IRC) of NMCTH (Reference Number: 57-074/075). The study was conducted between June and July 2018 and the whole sampling method was used. At the time of the study, there were 100 students enrolled in the first-year MBBS. Out of 100 questionnaires distributed, 91 were received.

Two sets of questionnaires were used in this study. The first set was a self-administered questionnaire focusing on sociodemographic factors concerning age, sex, living condition, and parent's education along with stressors like academic stress, hectic lifestyle, a broken relationship, and family problems. The second set of the questionnaire comprised of Depression, Anxiety, and Stress (DASS)-42 scale. Both of the questionnaires were pretested.

DASS is a self-report 42-item questionnaire developed by Lovibond and Lovibond proposed by the Australian Psychological Society to measure the emotional state of depression, anxiety, and stress. Each of the subscales contains 14 items and each item have a four-point severity scale “0” for “Did not apply to me at all”, “1” for “Applied to me to some degree”, “2” for “Applied to me considerable degree”, and “3” for “Applied to me very much". Scores for depression, anxiety, and stress are calculated by summating the score of the relevant item and it evaluates the severity of participant's experiences over the last week.^6^

Students were contacted and informed about the purpose of the study and explained about the questionnaire and asked to take part in the study. Informed consent was taken before the distribution of the questionnaire. Filling the questionnaire was completely voluntary and confidentiality was ensured. Data were calculated with Statistical Package for the Social Sciences (SPSS) Version 16. Descriptive statistics were applied and results were expressed in frequency and percentage.

DASS-42 scale only evaluates the participant experiences of the last seven days and hence might not reflect the actual mental state of medical students and the severity of experiences might differ considerably during the pre and post-exam period.

## RESULTS

The overall prevalence of Depression, Anxiety, and Stress among the first-year medical students was found to be 40 (44%), 54 (59.3%), and 41 (45.1%) respectively.

Out of 100 questionnaires distributed, 91 were received; the response rate was 91%. Among them, 49 (53.8%) were male and 42 (46.2%) were female. The mean age of the study participants was 19±1.32 years and range from 17 to 26 years. Concerning living conditions, 81 (89%) students lived in a hostel and 10 (11%) lived outside the hostel. About the education degree of the student's parents, 15 (16.5%) fathers were educated up to grade 10 and 76 (83.5%) of them were educated higher than grade 10. Similarly, 30 (33%) of the students' mothers were educated up to grade 10 and 61 (67%) were educated higher than grade 10.

Among various grades of depression, anxiety, and stress, the majority of students were found to be in the mild to moderate level. Very few students were in severe to extremely severe levels of depression, anxiety, and stress (Table 1).

**Table 1:** Levels of depression, anxiety, and stress among first-year medical students.

Levels	Depression n (%)	Anxiety n (%)	Stress n (%)
Normal	51 (56)	37 (40.7)	50 (54.9)
Mild to Moderate	29 (31.9)	33 (36.3)	33 (36.3)
Severe to Extremely Severe	11 (12.1)	21 (23.1)	8 (8.8)

The majority of students who had either depression, anxiety, or stress reported academic stress as their common stressor (Table 2).

**Table 2:** Sociodemographic characteristics and stressors of medical students with depression, anxiety, and stress.

	Variables	Depression (n = 40) n(%)	Anxiety (n = 54) n(%)	Stress (n = 41) n(%)
1	Gender			
	Female	19 (47.5)	27 (50)	20 (48.8)
	Male	21 (52.5)	27 (50)	21 (51.2)
2	Living conditions			
	Hostel	36 (90)	48 (88.9)	36 (87.8)
	Outside Hostel	4 (10)	6 (11.1)	5 (12.2)
3	Education level of Father			
	Educated up to grade 10	8 (20)	10 (18.5)	8 (19.5)
	Educated higher than grade 10	32 (80)	44 (81.5)	33 (80.5)
4	Education level of Mother			
	Educated up to grade 10	14 (35)	19 (35.2)	15 (36.6)
	Educated higher than grade 10	26 (65)	35 (64.8)	26 (63.4)
5	Academic stress			
	Yes	33 (82.5)	37 (68.5)	31 (75.6)
	No	7 (17.5)	17 (31.5)	10 (24.4)
6	Hectic lifestyle			
	Yes	14 (35)	17 (31.5)	14 (34.1)
	No	26 (65)	37 (68.5)	27 (65.9)
7	Broken relationship			
	Yes	9 (22.5)	8 (14.8)	8 (19.5)
	No	31 (77.5)	46 (85.2)	33 (80.5)
8	Family problems			
	Yes	4 (10)	4 (7.4)	3 (7.3)
	No	36 (90)	50 (92.6)	38 (92.7)

## DISCUSSION

In our study, the overall prevalence of depression, anxiety, and stress among first-year medical students were found to be 44%, 59.3%, and 45.1% respectively. A study conducted among first-year medical students in an Egyptian Public University reported the prevalence of depression, anxiety, and stress to be 63.6%, 78.4%, and 57.8% respectively which is higher compared to our study.^7^ On the other hand, a study conducted among medical students in two medical colleges in Nepal reported the prevalence of depression, anxiety, and stress to be 29.9%, 41.1%, and 27% respectively which is lower than the finding of our study.^8^

The burden of depression among medical students is significantly higher in Asian countries compared to a western study in the United States of America.^9^ These conflicting findings with our study may be due to differences in academic curriculum, help-seeking behavior, mental health awareness, and stress coping strategies among medical students.

In our study, students reported academic stress as one of the common stressors. This could indicate the mental health of medical students might improve after implementing a student-friendly curriculum, better learning techniques, and alternative ways to alleviate academic stress without hindering the objective of the medical curriculum. Two other studies conducted in Nepal among medical students also reported academic-related stressors as considerable distress among them.^10-11^

The limitation of our study is that it was conducted among first-year medical students of a single hospital and it cannot be generalized to all the first-year medical undergraduates of Nepal. There is no consideration of genetic predisposition, family history, pre-existing mental disorders of the medical students. Students were not assessed about their coping strategies. The recommendations would be to conduct a study in a large population multicentre setting with consideration of the above-mentioned factors.

## CONCLUSIONS

The result of the study elucidate the frequency of depression, anxiety, and stress was higher in the early years of medical school. It is recommended to implement screening programs including programs to teach positive self-coping techniques, assignment of professional counselors and mentors to help and identify students suffering from symptoms of depression, anxiety, and stress right from the start of medical school. It is important to address their problems early to avoid extreme unfortunate consequences such as dropouts and suicides. There should be the availability of counselors who are not involved in the academic education of medical students and who can provide a safe environment where students feel at ease to share their problems without feeling judged.
